# Implementation of cylindrical PET scanners with block detector geometry in STIR

**DOI:** 10.1186/s40658-019-0248-9

**Published:** 2019-07-29

**Authors:** Parisa Khateri, Jannis Fischer, Werner Lustermann, Charalampos Tsoumpas, Günther Dissertori

**Affiliations:** 1 Institute for Particle Physics and Astrophysics, Department of Physics, ETH Zürich, Zürich, Switzerland; 2 Leeds Institute of Cardiovascular and Metabolic Medicine, University of Leeds, Leeds, UK

**Keywords:** PET image reconstruction, SAFIR, Scanner geometry, STIR

## Abstract

**Background:**

Software for Tomographic Image Reconstruction (STIR) is an open-source library for PET and SPECT image reconstruction, implementing iterative reconstruction as well as 2D- and 3D-filtered back projection. Quantitative reconstruction of PET data requires the knowledge of the scanner geometry. Typical scanners, clinical as well as pre-clinical ones, use a block-type geometry. Several rectangular blocks of crystals are arranged into regular polygons. Multiple of such polygons are arranged along the scanner axis. However, the geometrical representation of a scanner provided by STIR is a cylinder made of rings of individual crystals equally distributed in axial and transaxial directions. The data of realistic scanners are projected onto such virtual scanners prior to image reconstruction. This results in reduced quality of the reconstructed image. In this study, we implemented the above-described block geometry into the STIR library, permitting the image reconstruction without the interpolation step. In order to evaluate the difference in image quality, we performed Monte Carlo simulation studies of three different scanner designs: two scanners with multiple crystals per block and one with a single crystal per block. Simulated data were reconstructed using the standard STIR method and the newly implemented block geometry.

**Results:**

Visual comparison between the images reconstructed by the two models for the block-type scanners shows that the new implementation enhances the image quality to the extent that the results before normalization correction are comparable with those after normalization correction. The simulation result of a uniform cylinder shows that the coefficient of variation decreases from 25.8% to 20.9% by using the new implementation in STIR. Spatial resolution is enhanced resulting in a lower partial loss of intensity in sources of small size, e.g., the spill-over ratio for spherical sources of 1.8 mm diameter is 0.19 in the block and 0.34 in the cylindrical model.

**Conclusions:**

Results indicate a significant improvement for the new model in comparison with the old one which mapped the polygonal geometry into a cylinder. The new implementation was tested and is available for use via the library of Swiss Federal Institute of Technology in Zurich (ETH).

## Background

Positron emission tomography (PET) allows quantitative imaging of the radiotracer’s distribution inside the object under study. There is a strong demand in pre-clinical PET imaging for accurate reconstruction procedure to acquire quantitative images of high spatial resolution. Iterative image reconstruction methods are especially of interest as they yield accurate quantitative images [1]. They can incorporate physical effects of the system into the reconstruction model and enhance the spatial resolution [2]. One of the fundamental components in iterative reconstruction algorithms is the system matrix which models the scanner response [3, 4]. Different methods have been developed to derive the system matrix. One method is to measure a point source located in different positions in the scanner field of view (FOV) [5]. Another approach is to obtain the system matrix using Monte Carlo simulation [6, 7]. Such simulations can include both geometrical and physical properties of the system but they are computationally challenging. An alternative is to analytically calculate the line of intersection between image voxels and lines of response (LOR) [8]. In the analytical approach, the system matrix can be decomposed into different components to take into account various resolution-degrading effects [9, 10], as follows:

1 $$ q= NLXf+s+r $$

**Fig. 1:**
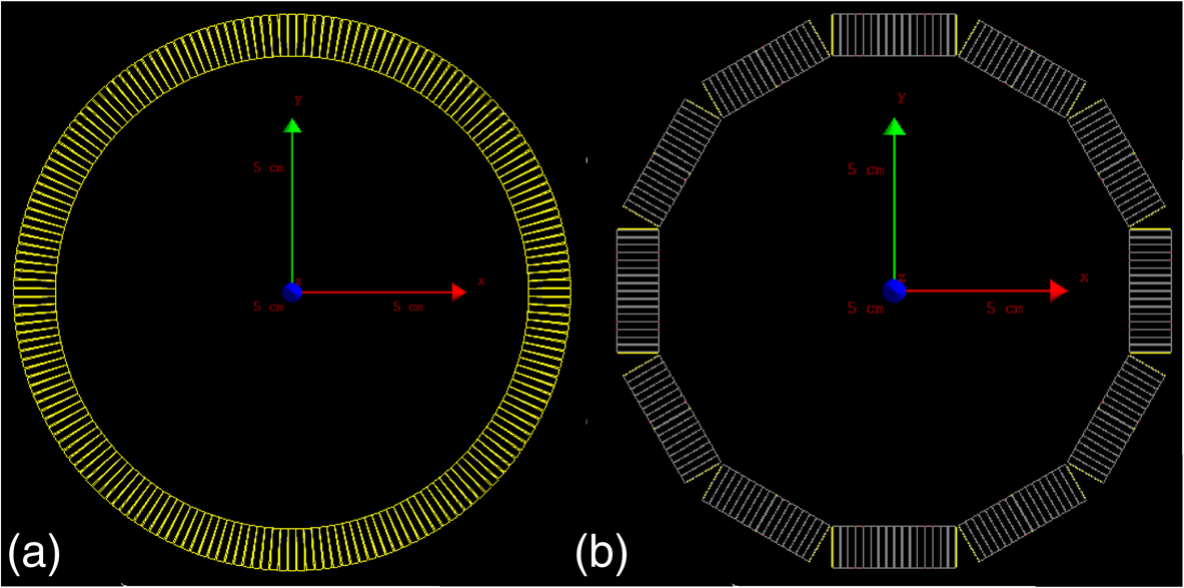
Schematics of scanner models. **a** Cylindrical geometry. **b** Block geometry

This equation is used as the forward model in the well-known expectation maximization algorithm [1, 11]. It describes the mean of the measured data, vector ***q***, as a function of the radiotracer’s distribution, vector ***f***. Diagonal matrices of *N* and *L* represent normalization and attenuation correction, respectively. Vectors of ***s*** and ***r*** are estimates of the mean of scatter and random events, respectively. Matrix *X* is the geometric model that relates the projection space to the image space. This geometric element is directly dependent on the model of the scanner geometry. In this study, we concentrate on this key component of the system matrix and in particular its implementation in STIR (Software for Tomographic Image Reconstruction) [12]. STIR is an open-source library for PET and SPECT image reconstruction, implementing analytical as well as iterative reconstruction. Within STIR, the system matrix is calculated based on the analytical method with the Siddon raytracing algorithm [13]. The geometrical representation of a scanner provided by STIR is a cylinder made of rings of individual crystals equally distributed in axial and transaxial directions. The data of realistic scanners are projected onto such virtual scanners prior to image reconstruction. Whereas, typical scanners, clinical as well as pre-clinical ones, use a block-type geometry. Several rectangular blocks of crystals are arranged into regular polygons. Multiple of such polygons are arranged, with equal spacing, along the scanner axis. Gaps between individual crystals within a block are typically different from the gaps between the blocks in axial and transaxial directions. The difference between these two models, in a transaxial direction, is schematically shown in Fig. 1.

We implemented the block geometry in both axial and transaxial directions in the STIR library and investigated if the implementation of such a realistic model is worth the time and computational expense. The new implemented model permits the image reconstruction without the interpolation step. Therefore, LORs can be described more accurately which in turn leads to more accurate localization of radiotracers. Details of the implementation along with the evaluation of the reconstruction are described in the next sections.

## Methods

### Software implementation

The new block geometry was implemented in STIR version 3.0. STIR makes use of the object-oriented features of C++. The library contains main building blocks including categorized classes to describe certain kinds of object with their functionalities, e.g., to describe images, 3D sinograms, forward/backward projection, iterative image reconstruction algorithms, and reading/writing interfile headers (I/O) [12]. We now describe these features in more detail.

The class *Scanner* describes the characteristics of a cylindrical scanner with discrete detectors. It lacks parameters to be able to model the exact block geometry, e.g., the distance between crystals in the axial and transaxial directions and the distance between blocks in the axial direction. We added these new parameters to the class *Scanner* and to the I/O-related classes accordingly. Moreover, the interfile headers were modified such that the user can choose at run-time which geometry to use, either *cylindrical* or *blocks-on-cylindrical*.

The library has been extended to be able to read custom input file formats, for instance, the Small Animal PET Insert For MRI (SAFIR) list-mode data for a cylindrical scanner, and to histogram them in 3D sinograms [14]. This part was adapted to cope with the new block geometry as well. In the cylindrical implementation, sinogram bin parameters are found from the LOR Cartesian coordinates. Bin parameters are segment, axial-position, view, and tangential-position numbers. We overloaded a function to calculate the sinogram bin parameters directly from the detector position numbers.

**Fig. 2:**
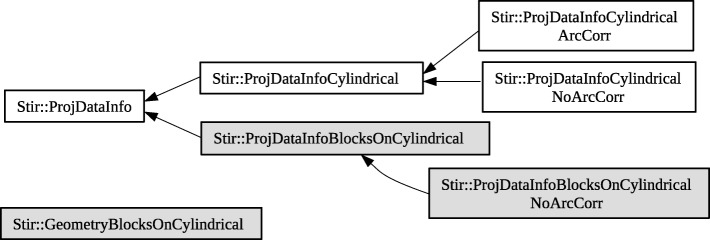
Class hierarchy related to sinogram data. Arrows indicate the parent class. Gray boxes show the new classes added to the library. The classes *ProjDataInfoBlocksOnCylindrical* and *ProjDataInfoBlocksOnCylindricalNoArcCorr* store and calculate the sinogram LOR and bin information for the block geometry. The class *GeometryBlocksOnCylindrical* builds a map of Cartesian coordinates of detectors according to the scanner geometry

The most important building block to modify was the one related to sinograms and forward/backward projection. New classes were inherited in which the 3D-sinogram information could be calculated according to the new scanner geometry. Figure 2 demonstrates the related class hierarchy. In the former model, the LOR coordinates are calculated based on detector position numbers with the assumption of having a perfect cylindrical scanner. In the current implementation, we calculate LOR coordinates from the exact Cartesian coordinates of the two detection positions. The detection positions are calculated based on the average depth of interaction inside the crystals. In both geometries, it is possible to use LOR sampling in a tangential direction. It means that multiple rays could be used in raytracing for each detector pair. In this case, the spatial sampling is evenly distributed and the result is averaged over different LORs.

### Monte Carlo simulations

We simulated three sets of PET data with different scanner designs using Geant4 toolkit v10.2 [15] and Gate v7.2 [16]: a scanner with a single crystal per block to test the code implementation and two scanners with multiple crystals per block but different crystal sizes to evaluate the new model. Details of the simulation setup are described as follows.

#### The scanner with a single crystal per block—Scanner A

This scanner, simulated using Geant4 toolkit, is built of 41 rings evenly distributed in axial direction comprising 180 detector elements uniformly distributed per ring. The crystal size is 2.0 × 2.0 × 12.0 mm^3^, and the inner ring radius is 63.02 mm. The crystal pitches in axial and transaxial directions are 2.2 mm.

A Derenzo phantom with spherical sources of 1.0, 1.2, 1.6, 2.4, 3.2, and 4.0 mm diameter was simulated inside this scanner. The spheres were filled with 500 MBq of ^18^F and located inside a water cylinder with 25 mm diameter. The activity was distributed uniformly inside the spheres. Simulation was performed for 5 s.

#### The block-type scanner with 2.1 × 2.1 × 12 mm^3^ crystals—Scanner B

The geometry of this scanner is similar to the SAFIR prototype scanner [17]. It is composed of 2 (24) blocks of 8 × 8 detectors in axial (transaxial) direction. The blocks are arranged on a dodecagon prism, i.e., there are two blocks per module, each module on one side of the prism. The inner radius of the scanner is 67.75 mm. The crystal size is 2.1 × 2.1 × 12.0 mm^3^. Crystal gap and block gap are 0.1 mm and 0.6 mm, respectively, in both axial and transaxial directions.

Several phantoms were simulated inside this scanner using Gate v7.2. The first one was a rotating plane (32 mm × 120 mm) source simulated to find the normalization correction factors. The plane was rotated at six equally spaced angles over a total of 180°. Since the scanner has 12 modules of detector blocks, this assures that the number of detected LORs, which are orthogonal to the plane, is maximized. The source activity was set to 10 MBq at each angular position. The acquisition time was 80 min per position. We used back-to-back emission of two 511-keV gammas, thus, the non-collinearity and positron range was not simulated in this set of data. The plane was filled with air to avoid scatter and attenuation effects. The decay was turned off. This increases the statistics per detector pair and therefore reduces the statistical error introduced by the normalization.

A uniform water-filled cylinder (25 mm × 60 mm Ø) which contained 100 MBq of ^18^F was simulated for 2.5 min.

A point source consisting of 10 MBq of ^22^Na was embedded inside an acrylic cube of 10.0 mm extended on all sides as suggested in NEMA NU 4 [18]. The source was located at the axial center and one-fourth of the axial FOV. For both axial positions, the source was moved radially at positions of 0.0, 5.0, 10.0, 15.0, 20.0, 25.0 mm. 10^5^ prompts were stored for each position.

A Derenzo phantom with sphere diameters of 1.6, 1.8, 2.0, 2.2, 2.4, and 2.8 mm made of water was used in order to evaluate the effect of spatial resolution on the image quality. The initial activity of 100 MBq of ^18^F was distributed uniformly inside the spheres, with the same activity per volume in the spheres. The acquisition time was 5 s. The spheres were located inside a water cylinder with 25 mm length and 30 mm diameter. Therefore, the effect of positron range was present in the simulation.

The image quality phantom as described in NEMA NU 4 [18] consisting of 3.7 MBq of ^18^F was simulated for 20 min. The image quality phantom has two parts. One part of 20 mm inner length includes five hot rods of different diameters (1.0, 2.0, 3.0, 4.0, and 5.0 mm) inside a polymethylmethacrylate (PMMA) cylinder. The other part of 30 mm inner length comprises of two cold rods of the same diameter (8.0 mm) inside a hot cylinder. Since the scanner is not long enough to cover the whole phantom, we had to repeat the same simulation with two different axial positions of the phantom so that in each simulation, one part is axially centered in the scanner.

#### The block-type scanner with 1 × 1 × 10 mm^3^ crystals—Scanner C

In order to validate if the results are independent of the crystal size, a similar block-type scanner with smaller crystals (1.0 × 1.0 × 10.0 mm^3^) was simulated using Gate v7.2. The scanner is made of 2 (24) blocks of 16 × 16 detectors in an axial (transaxial) direction where the blocks are arranged on a dodecagon prism. The inner radius of the scanner is 67 mm. Cristal gap and block gap are 0.1 and 0.3, respectively.

A Derenzo phantom, with spheres of 0.6, 0.8, 1.0, 1.2, 1.5, and 2.0 mm diameter made of water was placed inside the scanner. The spheres were uniformly filled by the total activity of 100 MBq of ^18^F and were simulated for 5 s. The spheres were located inside a water cylinder with 25 mm length and 30 mm diameter.

### Sorting coincidence data

The coincidence data were sorted using a coincidence time window of 1 ns and an energy window from 350 keV to 650 keV which is common in preclinical studies. Random events were removed from simulated data by checking the event history in the Monte Carlo simulation data.

List-mode simulated data were histogrammed into 3D sinograms of types block and cylindrical. Each sinogram bin represents one LOR, i.e., no compression was implemented in axial and transaxial directions. The sizes of sinogram data for the three scanners are summarized in Table 1.

**Table 1:** Size of sinograms and images with their dimensions for the three different scanners

Scanner	Size of sinograms	1Number of sinograms	Number of image voxels	Image voxel size (mm^3^)
Scanner A	90 × 60	1681	127 × 127 × 81	0.55 × 0.55 × 1.1
Scanner B	96 × 100	256	129 × 129 × 31	0.55 × 0.55 × 1.1
Scanner C	192 × 100	1024	213 × 213 × 63	0.257 × 0.257 × 0.55

### Normalization

Normalization factors were only calculated for the *Scanner B*. The method described by Bailey et al. [19] was adapted for this purpose in the following manner. Six data sets were acquired from six angular positions of the plane. The goal was to extract the LORs that were almost orthogonal to the plane source at each position. We extracted 16 almost orthogonal views per position. Therefore, all views were covered because there were 96 views in total. The extracted views were assembled to build a complete 3D sinogram. The symmetric views and axial positions were added together to increase the statistics per sinogram bins. The average number of counts per sinogram bin was about 1000. This gives a statistical error of ~ 3.2%. The bin values were then inverted to calculate the normalization factors. The sinograms were trimmed to 160 tangential positions to remove bins out of the desired FOV.

### Attenuation correction

Generation of the attenuation maps was quite straightforward since the data were from Monte Carlo simulation and we knew the exact phantom geometry and the material. The attenuation coefficient used for water was 0.096 cm^−1^ at 511 keV [20]. The attenuation correction sinograms were calculated from the exponential of the forward projection of the attenuation maps for both cylindrical and block geometries.

### Scatter correction

The scatter estimation was performed using the current implementation of single scatter simulation (SSS) algorithm in STIR [21] where the Klein-Nishina cross-section is used to calculate the probability of Compton scattering as a function of scattering angle. We calculated the down-sampled single scatter sinograms given: (i) the activity image which was reconstructed from non-scatter corrected data, (ii) the attenuation map, (iii) the attenuation map down-sampled with a factor of two, (iii) the scanner down-sampled with a factor of two in axial and transaxial directions keeping the same inner radius, (iv) the energy resolution of 20% similar to the simulation setup, (v) and the energy window of 350–650 keV. Then the result was up-sampled and fit to the activity image using Trispline interpolation. The down-sampling speeds up the process while it does not affect the final result significantly as the scatter distribution varies slowly with the detector pair.

### Reconstruction

Emission data were reconstructed using fully 3D-OSEM (ordered subsets expectation maximization) algorithm [22]. The number of subsets was set to six for the Scanners B and C and to five for the *Scanner A*. The number of subiterations was 24 for all reconstructions. STIR is able to compute the Poisson log-likelihood and its gradient on distributed computing platforms using the Message Passing Interface (MPI). We ran the reconstruction on 16 Intel cores on one compute node (2 × Intel® Xeon® EP E5-2660 v2 (Ivy Bridge) at 2.2 GHz (10 cores/socket)) from Mönch cluster at the Swiss National Supercomputing Centre. The compute node had 32 GB of 1600 MHz DDR3 RAM. The nominal frequency of the CPU was 2.2 GHz.

All ring differences were used in the reconstruction. The number of rays in tangential direction for raytracing each LOR was 10 for both block and cylindrical geometries. For the data from *Scanners B* and *C*, attenuation and scatter correction factors were included in the standard factorized system matrix. The data from the *Scanner B* were also corrected for normalization using the normalization factors calculated in the “Normalization” section. The number of voxels in each direction and the voxel size for the three different scanners are summarized in Table 1.

### Evaluation

A cylindrical region of interest (ROI) was drawn so that it covered the whole uniform cylinder phantom. The coefficient of variation (COV) was calculated as the ratio of the standard deviation (STD) to the mean of this ROI, similar to NEMA NU 4 [18] to evaluate the uniformity.

Line profiles were generated along spheres with a different diameter in Derenzo phantoms. The Derenzo data taken by the *Scanner B* were evaluated in more details in terms of peak-to-valley (PTV) and spill-over (SOR) ratios for both cylindrical and block models. Two ROIs were drawn around each individual sphere in the Derenzo phantom. One of them was a spherical ROI as large as the sphere itself, and the other an annular ROI with the inner diameter as large as the sphere diameter and the outer diameter twice the sphere diameter (Fig. 10). The SOR was calculated as the ratio of the mean of the annular ROI to the mean of the spherical ROI.

The recovery coefficient (RC) as well as the percentage of its standard deviation (%STD) were calculated for the hot rods in the image quality phantom as described in NEMA NU 4 [18].

The cylindrical and block models were also evaluated in terms of time and memory consumption. For this purpose, the computations were performed on a single core of a local computer (Intel® Core™ i7-4710MQ CPU at 2.50 GHz × 8). Single forward and backward projections as well as iterative OSEM reconstruction were implemented on a data set from the *Scanner B* with different sizes of the system matrix. In order to create different sizes for the system matrix, maximum ring difference of the 3D sinogram was changed from 0 to 15 by steps of 5. The number of subsets and subiterations for the OSEM algorithm were six and 12, respectively. The OSEM reconstruction was run without using the MPI option and with caching the system matrix enabled. Currently, the standard cylindrical model utilized symmetries existing in the system matrix in axial and transaxial directions. The number of symmetries in the block geometry is smaller than the cylindrical geometry. Only axial symmetries have been implemented for the block model in the new version.

## Results

### The scanner with a single crystal per block—Scanner A

**Fig. 3:**
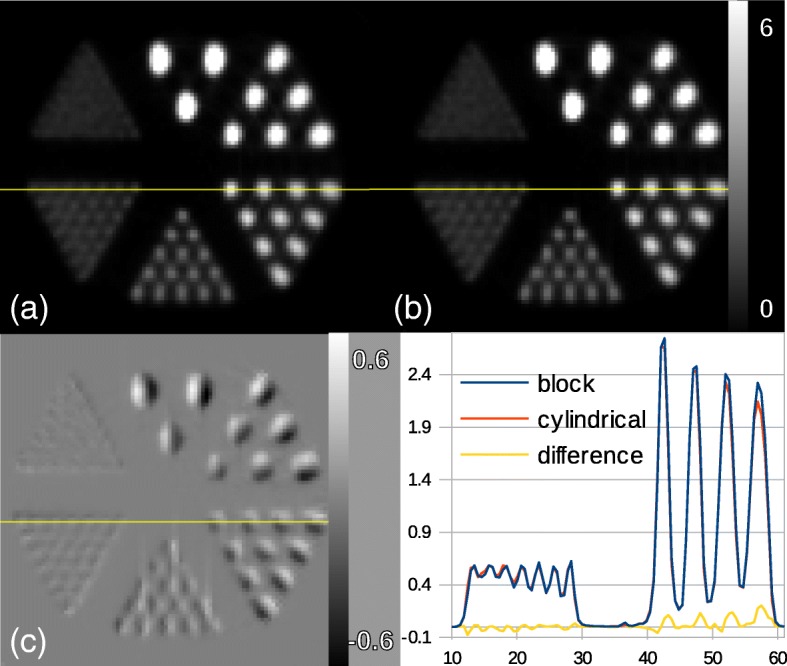
Images of the Derenzo phantom simulated inside the *Scanner A* reconstructed using OSEM algorithm with 5 subsets and 24 subiterations. **a** Cylindrical geometry, **b** block geometry, and **c** difference image as cylindrical subtracted from block. At the bottom-right, the line profiles through the sources of 1.2 mm and 2.4 mm diameter are plotted as well as the difference between the two line profiles. The color bar in the first row is common to both images

The comparison of the new implementation with the standard cylindrical implementation in STIR was performed without correcting data prior or during image reconstruction. The results from block and cylindrical model show equal reconstruction performance, however, not identical (Fig. 3). The reason is that the cylindrical model even for such a scanner design uses approximation to calculate LOR coordinates given sinogram bins. For instance, it assigns the same azimuthal angle to two tangentially adjacent LORs with slightly different azimuthal angles. The difference image in Fig. 3c illustrates this rotational difference between the images. The line profile plotted across images indicates the difference between voxel values across the line. Maximum relative difference between the images calculated over a cylindrical ROI (6 mm × 50 mm Ø) was 10%.

### The block-type scanner with 2.1 × 2.1 × 12 mm^3^ crystals—Scanner B

**Fig. 4:**
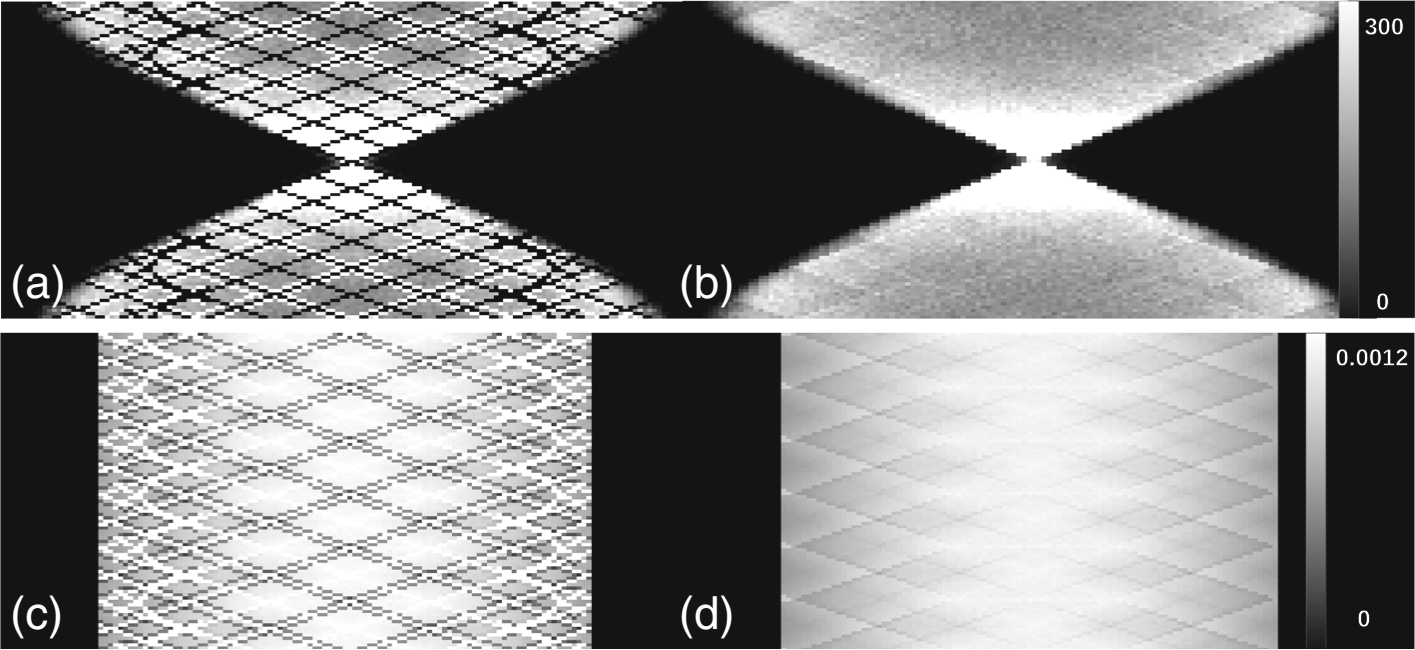
Direct sinograms in the center of the *Scanner B.* Rows and columns of the sinograms represent views and tangential positions, respectively. **a** Sinogram of the plane source located perpendicular to *y*-axis in cylindrical geometry, **b** Sinogram of the plane source located perpendicular to *y*-axis in block geometry, **c** normalization factors’ sinogram in cylindrical geometry, and **d** normalization factors’ sinogram in block geometry. The color bar in each row is common to both sinograms. However, for the cylindrical geometry, there are values which are more than the maximum value in the color bar. These values are depicted in white. Therefore in the sinogram (**a**), bins are greater than or equal to 300, and in the sinogram (**c**), bins greater than or equal to 0.0012 are white

For each angular position of the plane source, we simulated about 4.175 × 10^15^ counts in total. The maximum number of counts per sinogram bin for the block model was about 8.3 × 10^3^ and for the cylindrical one about 2 × 10^4^. Figure 4 shows direct sinograms from the plane source located perpendicular to the *y*-axis. Some of the bin values in the cylindrical model are always zero since no coincidence events fell into these bins. This is due to mapping the block-type scanner into a virtual cylindrical scanner. Normalization factors were calculated and histogrammed in sinogram bins. The zero bins in the measured sinograms of the cylindrical model have the highest intensity in the normalization sinograms as shown in Fig. 4c. The reason is because of the way the normalization sinograms are calculated, as inversions of the measured data. With a measured value of zero, we obtain the normalization value of infinity which is replaced in practice by a maximum value.

**Fig. 5:**
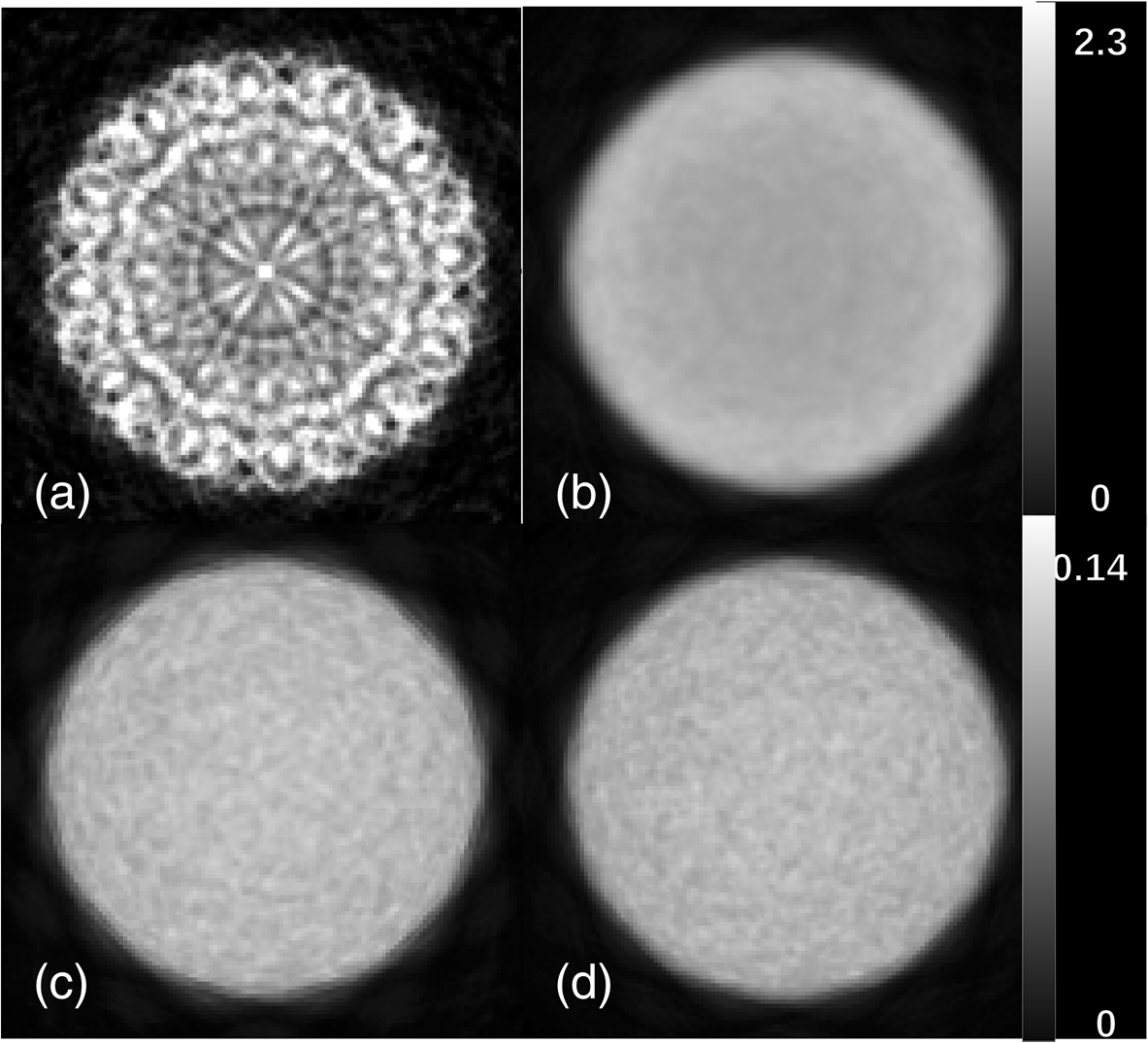
Images of the uniform cylinder phantom simulated inside the *Scanner B* reconstructed using OSEM algorithm with six subsets and 24 subiterations. **a** Cylindrical geometry without normalization, **b** block geometry without normalization, **c** cylindrical geometry with normalization, and **d** block geometry with normalization. The color bar in each row is common

The reconstruction was performed using both scanner models. Figure 5 shows the reconstructed images of the uniform cylinder phantom. The cylindrical model creates significant artifacts prior to normalization correction while the block model yields a smooth cylinder. The COV ratio for the block model is much smaller than the one for the cylindrical model (Table 2). After correcting for normalization, the block and cylindrical models show similar results. The COV ratio for the block model increases from 18.4% to 20.9%. Although, the block model is visibly more uniform than the cylindrical one after normalization. This can be inferred from the COV ratios in Table 2 which shows the COV ratio for the block model is 19% better comparing to the cylindrical one, after normalization.

**Table 2:** The COV ratio for the uniform cylinder phantom

Model	COV
Cylindrical without normalization	33.2%
Block without normalization	18.4%
Cylindrical with normalization	25.8%
Block with normalization	20.9%

**Fig. 6:**
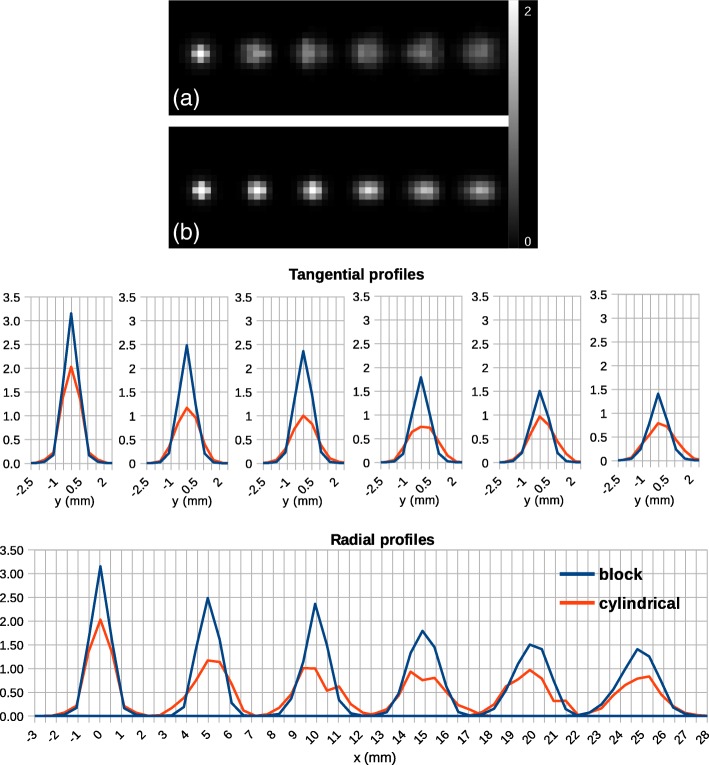
Images and line profiles of the point source simulated inside the *Scanner B* reconstructed using OSEM algorithm with six subsets and 24 subiterations. **a** Cylindrical geometry. **b** Block geometry. The top (bottom) row of line profiles is tangentially (radially) drawn across the point source. The position of the point source in the top row corresponds to those in the bottom row. The point source located at the center of the scanner moves radially till 25 mm with steps of 5 mm

**Fig. 7:**
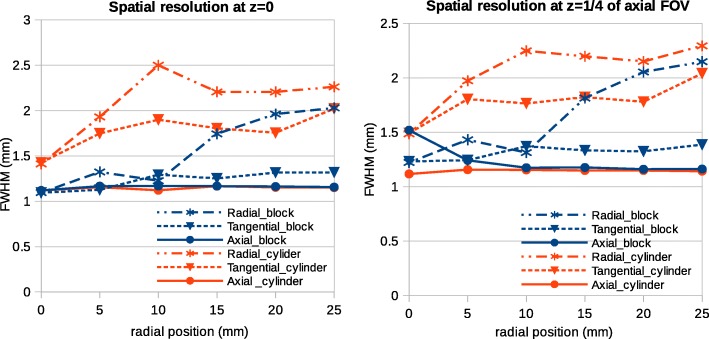
FWHM measurements for the point simulated inside the *Scanner B* reconstructed using OSEM algorithm with six subsets and 24 subiterations. The point source is axially at two different positions: the center of the scanner and one-fourth of the axial FOV. For both cases, it moves radially till 25 mm with steps of 5 mm. The FWHM values are plotted for three axial, tangential and radial directions for the block and cylindrical models

The results for the spatial resolution test are shown in Figs. 6 and **7**. Reconstructed images of the point source together with the radial and tangential line profiles indicate that the new implementation yields a sharper image with better localization of the point source (Fig. 6). For the point at 15 mm, the radial and tangential line profiles of the new implementation are very close to the ones of the standard cylindrical implementation. Figure 7 shows the full width half maximum (FWHM) in radial, tangential, and axial directions measured for the two axial positions: at the center and at one-fourth of the axial FOV. Both axial positions show a similar pattern. Radial and tangential values of FWHM are significantly larger for the cylindrical model. In a radial direction, FWHM increases by moving to the edge of the scanner which is reasonable as the point radially moves to the edge. In the axial direction, the two models show almost the same FWHM except for the point radially centered and axially located at 8.9 mm from the center of FOV. The axial FWHMs are 1.5 mm and 1.2 mm, respectively, for the block and the cylindrical implementations. This could be due to the discretization artifact as for the axially centered position; the two models show similar results.

**Fig. 8:**
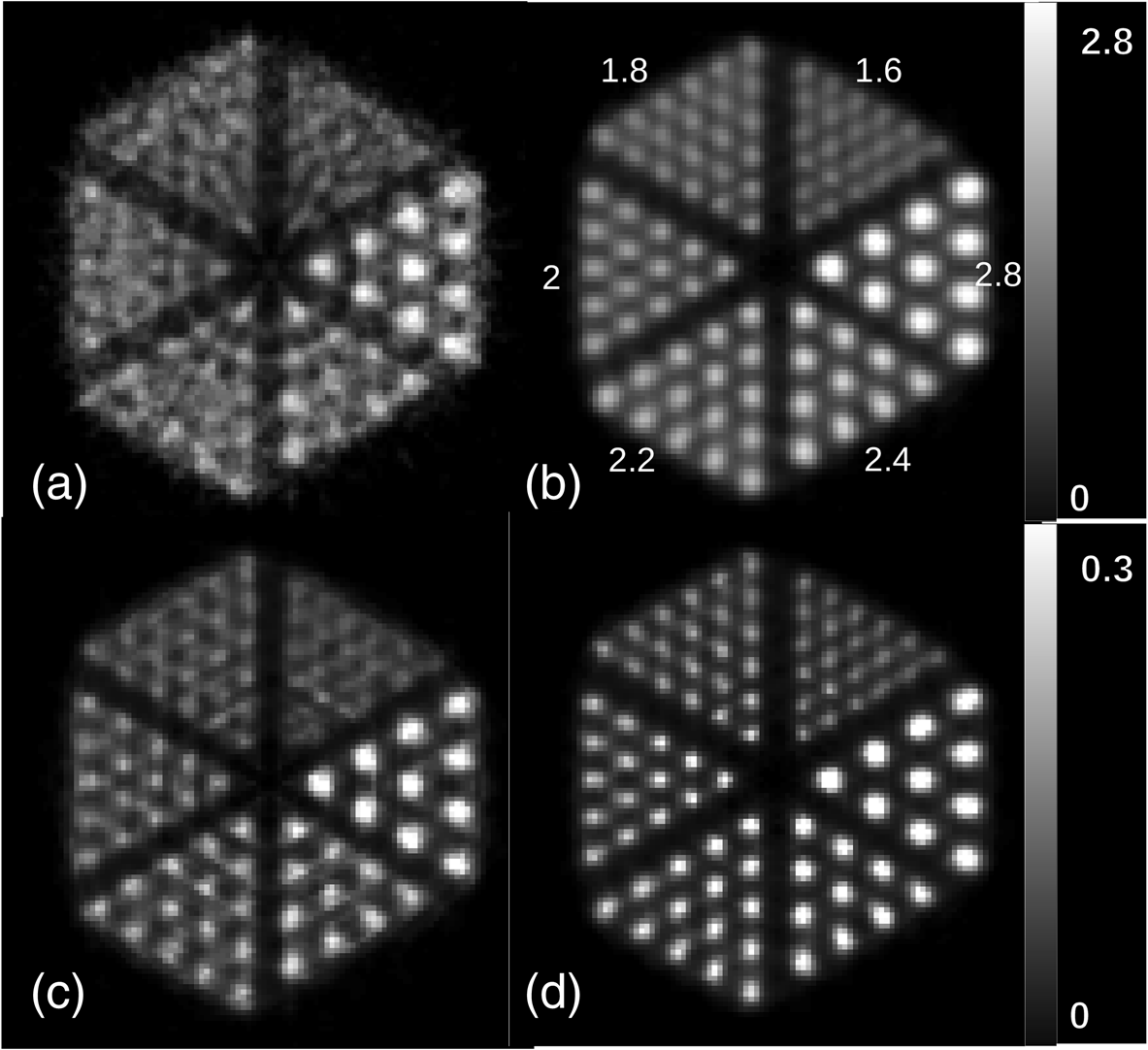
Images of the Derenzo phantom simulated inside the *Scanner B* reconstructed using OSEM algorithm with six subsets and 24 subiterations. **a** Cylindrical geometry without normalization, **b** block geometry without normalization, **c** cylindrical geometry with normalization, and **d** block geometry with normalization. A circular artifact is observed in images **a** and **c**. Values on image **b** show the sphere diameters in millimeter

Visual comparison between the reconstructed images of the Derenzo phantom indicates a significant improvement for the block model (Fig. 8). Smallest spheres of 1.6 mm diameter can be clearly resolved using the new model even without applying normalization. Whereas, it is not possible to distinguish between some sources of 1.6 mm diameter in the cylindrical model after applying normalization. A circular artifact is observed in Fig. 8a which stays after normalization in Fig. 8c.

**Fig. 9:**
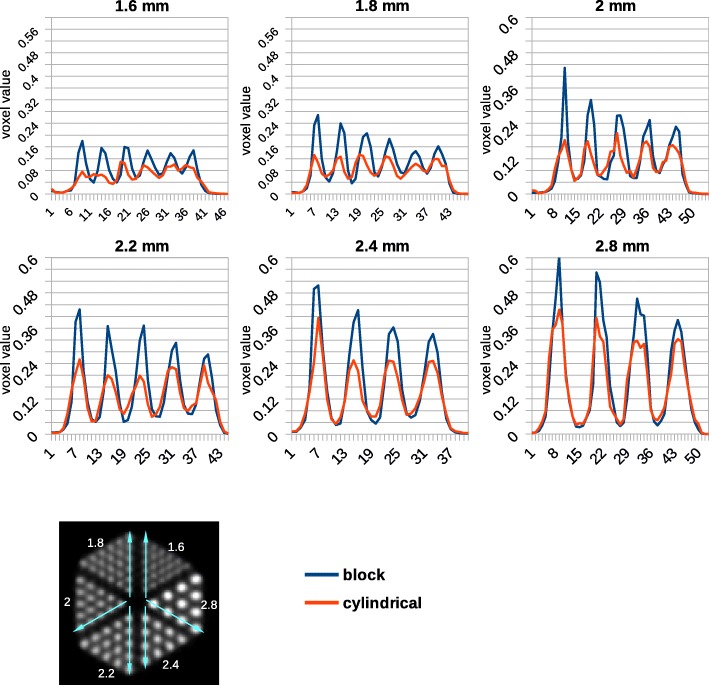
Line profiles across images of the Derenzo phantom simulated inside the *Scanner B* reconstructed using OSEM algorithm with six subsets and 24 subiterations with normalization. Each graph represents a different sphere size which is written on top of the graph. A schematic of line profiles is drawn at the bottom

Line profiles across spherical sources with different diameters are plotted in Fig. 9. The average PTV ratio for these line profiles is higher for the block model (Fig. 11). It is almost twice the cylindrical model, e.g., 3.63 and 2.07 for the 1.8 mm spheres, respectively. Line profiles indicate that the PTV ratio decreases from the peripheral area to the center of the phantom. It also decreases by decreasing the source size.

**Fig. 10:**
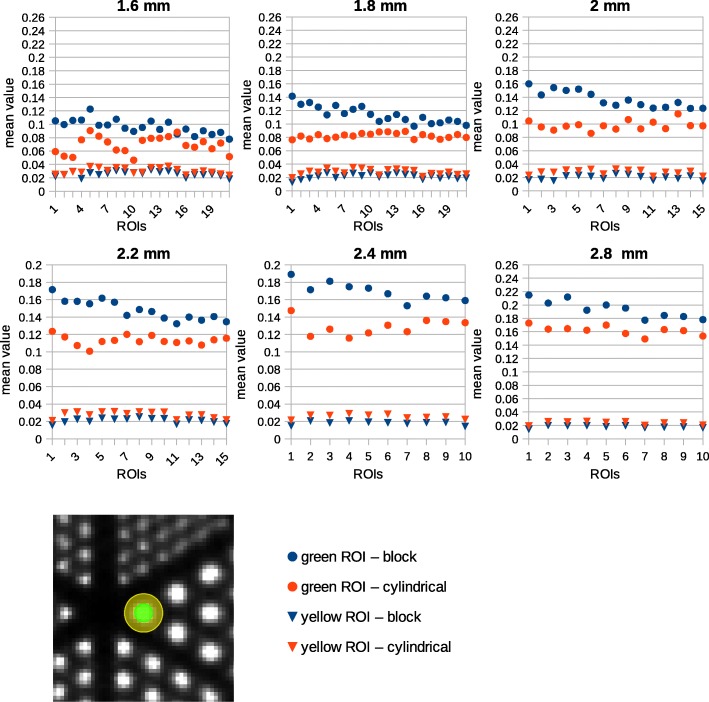
Mean value for spherical and annular ROIs around all sources in the Derenzo image. Each graph represents a specific sphere size which is shown on top of the graph. The spherical ROI and the annular ROI are depicted by green and yellow, respectively, in the image in the third row

**Fig. 11:**
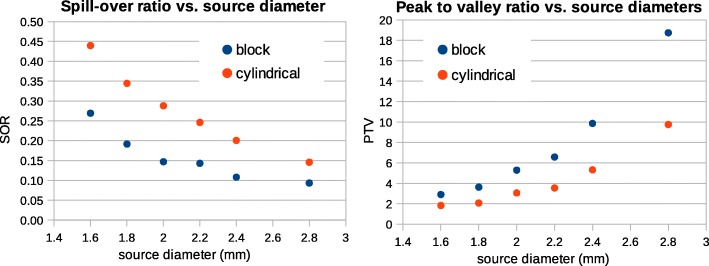
SOR and PTV calculated for different source size in Derenzo phantom after normalization correction

Figure 10 indicates the mean value for spherical and annular ROIs in the Derenzo images. The values of spherical ROIs are higher for the block and those of the annular ROIs are higher for the cylindrical model. This implies a lower partial loss of intensity for the new model. The SOR of 1.8-mm spheres is 0.19 and 0.34 for the block and cylindrical geometries, respectively. This value increases by increasing the source size (Fig. 11). There is a little kink in the SOR graph at 2.2 mm. The reason is that the spheres of 2.2 mm diameter are more extended to the peripheral area of the field of view where the spatial resolution degrades and the partial volume effect is more visible.

**Fig. 12:**
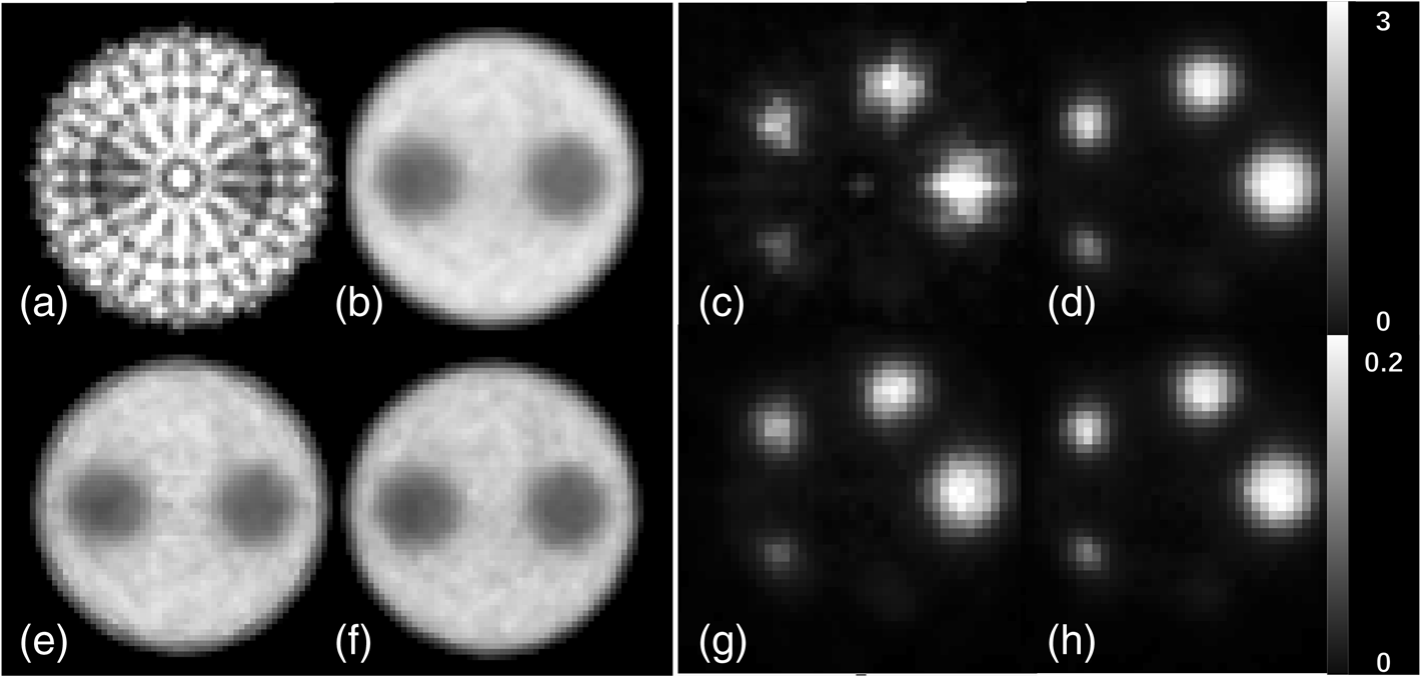
Images of the image quality phantom simulated inside the *Scanner B* reconstructed using OSEM algorithm with six subsets and 24 subiterations. Transverse planes through the part with the cold rods: **a** cylindrical geometry without normalization, **b** block geometry without normalization, **e** cylindrical geometry with normalization, and **f** block geometry with normalization. Transverse planes through the part with the hot rods: **c** cylindrical geometry without normalization, **d** block geometry without normalization, **g** cylindrical geometry with normalization, and **h** block geometry with normalization. The images are transverse planes through the part containing hot rods. The color bar in each row is common

**Fig. 13:**
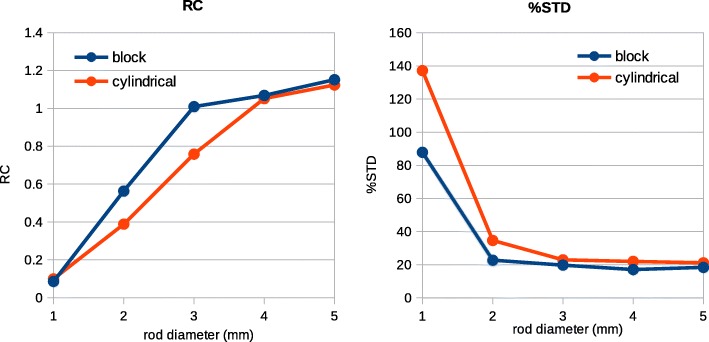
RC values together with their %STD for hot rods of different diameter in the image quality phantom

Similar to the uniform cylinder and the Derenzo phantoms, both parts of the image quality phantom show that the new model enhances the quality of the images (Fig. 12). The part with cold rods inside the hot cylinder shows similar artifact as the uniform cylinder phantom before normalization (Fig. 12a). After normalization, the two models visually show similar results. As for the hot rods inside the PMMA cylinder, it could be observed in Fig. 12c that the cylindrical model tends to intensify the central region. This is because the density of the LORs in the center for the virtual cylindrical model is higher than reality. The RC values as well as their %STDs are plotted in Fig. 13 showing a higher RC and lower %STD for the block model. The RC values are especially higher for the rods of 2 mm and 3 mm diameters.

### The block-type scanner with 1 × 1 × 10 mm^3^ crystals—Scanner C

**Fig. 14:**
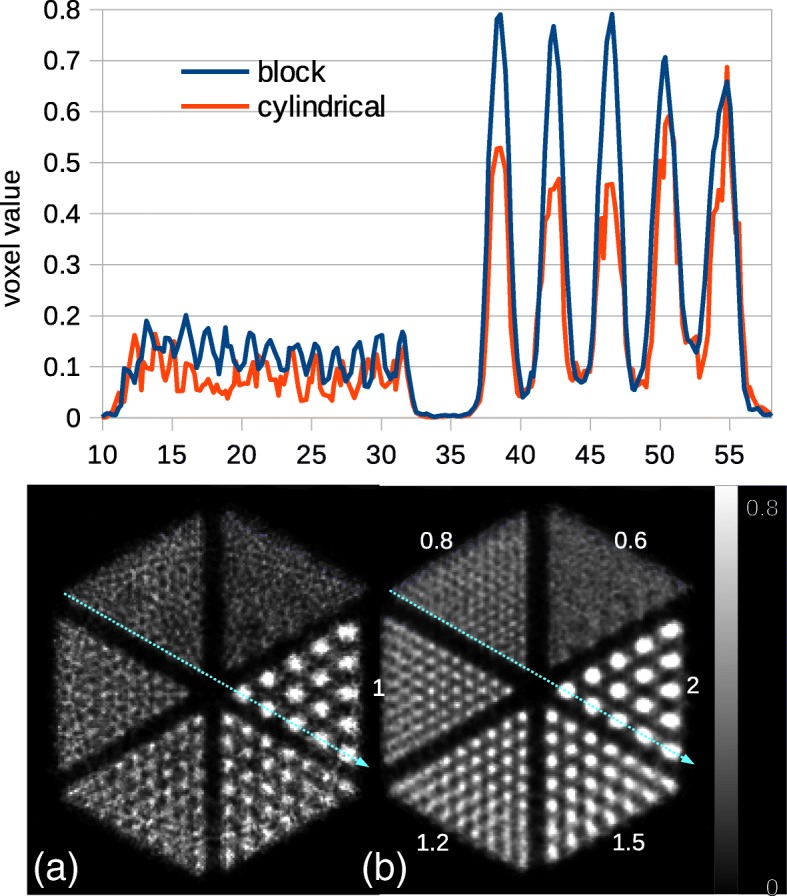
Images of the Derenzo phantom simulated inside the *Scanner C* reconstructed using OSEM algorithm with six subsets and 24 subiterations. **a** Cylindrical geometry. **b** Block geometry. The images are not normalized. At the top, the line profiles through the sources of 0.8 mm and 2 mm diameter are plotted for the two models. Values on image **b** show the sphere diameters in millimeter

Reconstructed images of the Derenzo phantom with spheres of 0.6 mm diameter simulated inside the *Scanner C* are demonstrated in Fig. 14. These results are without normalization correction. It can be seen that the new implementation enhances the image quality. The result from block model can resolve most spheres down to 0.8 mm diameter without normalization correction. However, neither of two models can distinguish between the smallest spheres which are about half the crystal pitch. The line profile across spheres of 0.8 mm and 2 mm diameter for both models indicates that the new model is less noisy comparing to the standard cylindrical model. The goal of this measurement was only to show that the new implementation works with smaller crystal size. Therefore, we did not perform the evaluation procedure as for the *Scanner B*.

### Time and memory consumption in the two models

**Fig. 15:**
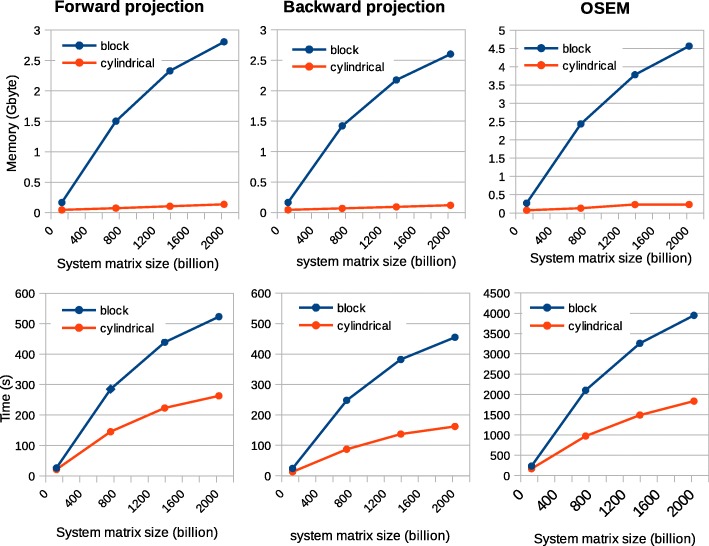
Time and memory consumption for different algorithms vs. the size of the system matrix for the two models. The top (bottom) row shows the memory (time) consumption. The first, second, and third columns represent the forward projection, backward projection, and OSEM algorithm with six subsets and 12 subiterations. Each plot consists of four different sizes of the system matrix which correspond to different maximum ring differences of 0, 5, 10, and 15

Figure 15 summarizes the time and memory consumption versus the size of the system matrix. The measurements have been done for the two models for different algorithms, forward and backward projection as well as OESM reconstruction. The memory usage of the three processes does not vary so much for each model. However, the block model consumes much more comparing to the cylindrical one especially by increasing the size of the system matrix. For the OSEM algorithm with six subsets and 12 subiterations, the memory usage reaches 4.5 GB for the block model comparing to 0.25 GB for the cylindrical model. The increase of time is less significant than the memory consumption for the block mode comparing to the cylindrical model. For the largest system matrix, the OSEM algorithm lasts about 65 min for the block model while it is about 28 min for the cylindrical model.

## Discussion

A precise scanner model is essential to build a precise system matrix in the PET reconstruction. Simplified assumptions could cause wrong LOR coordinates and therefore wrong positioning of the source distribution. The effect of remapping the scanner into a virtual cylindrical scanner varies for different scanner geometries. When STIR operates in the cylindrical mode, it maps the block-type scanner on a cylinder with the same inner radius. This means LOR coordinates in the cylindrical mode are slightly different compared to the block mode. For a given LOR—described with azimuthal angle, polar angle, axial position, and tangential position—STIR tries to find the corresponding sinogram bin. Since detector positions in the cylindrical mode are rearranged for a block-type scanner, LORs could be assigned to wrong bins. Some bins might be chosen more often than in the correct geometry and some bins might be never chosen (zero bins in Fig. 4a). Consequently, also the maximum number of counts per sinogram bin is different for the two models. This could be especially observed in the results for the rotating plane source which has a high statistics (Fig. 4).

Using the more accurate block model, we expect to get smoother sinograms for the plane source (Fig. 4b), however, still not perfectly smooth. A slight non-uniformity is observed close to the edges of the detector blocks in the sinogram of the block model. This effect is well pronounced in Fig. 4d in the normalization sinogram which is the result of the superposition of the measured sinograms followed by an inversion. This slight non-uniformity, unlike the severe non-uniformity in the cylindrical model, is because of the crystal interference effect within a block. It is a pattern that is repeated for the detectors in every block [19].

The normalization corrects the non-uniformity caused by geometric effects and the non-uniformity of detector responses. The latter does not exist in our simulations, and since the new implementation in STIR can more accurately model the block-type scanner, the non-uniformity in the sinogram and consequently in the image is reduced. Figure 5b indicates that the block model produces a uniform cylinder even before normalization. However, there is a small residual of non-uniformity that could be observed in the middle of the cylinder which disappear after normalization (Fig. 5d).

On the other hand, the result of COV indicates that applying normalization correction introduces a noise into the image reconstructed by the block model. The reason is the limited statistics per LOR. Equation (1) shows that the uncertainty of a given voxel value increases corresponding to the number of bins contributing in calculation of that voxel, as well as the uncertainty of the normalization factors (3.2%). By increasing the statistics, the uncertainty in normalization factors decreases, and therefore, the noise introduced by normalization decreases. As an alternative, component-based methods could be used to find normalization factors with lower statistics. To investigate the effect of statistics on the image uniformity, we calculated the normalization factors with one-tenth of the current statistics. The reconstructed images using these normalization factors are much noisier. The COV for the lower statistics case is 42.8% compared to 20.9% calculated for the higher statistics case.

In the Monte Carlo simulation, we did not simulate the non-uniformity of the detector responses, which in reality can add to the image noise. This means, in the real scanner, the proper normalization is essential to improve the uniformity even in the case of block implementation. Additional studies can be performed to assess the block model with real data acquired from different scanner designs such as the SAFIR scanner.

As for the spatial resolution, the simulation results show that the new model improves the images compared to the standard cylindrical model. The line profiles across the point sources show that in the transaxial plane, the spatial source distribution is much sharper for the block model, while in the axial direction, the two models give similar results. The reason is that the difference between the two models in the axial direction is only a gap between the two detector blocks while in the transaxial plane, the difference is more significant because all detector elements are slightly dispositioned for the cylindrical model.

The results from Derenzo phantom (Fig. 8) show that although normalization can improve the quality of the image for the cylindrical implementation, it still leaves artifacts in the reconstructed image. The normalized Derenzo image reconstructed with the cylindrical model in Fig. 8c shows a circular artifact.

In the Derenzo phantom, it may look like the image intensity is higher in the center of the phantom, but this is not the case, because the average intensity stays the same across larger regions in the phantom. This can be inferred from the line profiles where the peak values decrease to the peripheral area but the valley values increase. This is due to the spill-over effect that is caused by degraded spatial resolution in the peripheral area. The SOR ratio is another evidence for this effect since it increases at the center of the phantom.

RC values calculated for the image quality phantom show that the block model can better recover the intensity for the rods of 2 mm and 3 mm diameter. The block model decreases the partial volume effect and enhances the image contrast where the source size is close to the size of detector elements. For larger rods, the two models give similar results. The RC value for the smallest rod is not reliable due to the high %STD which is 90% and 140%, respectively, for the block and cylindrical model. The 1-mm rod is out of the resolution of the *Scanner B*.

The use of more accurate block geometry is computationally more intensive as the number of symmetries in the block geometry is smaller than the cylindrical geometry. This means that more calculations are required for the system matrix elements in each forward/backward projection in the iterative reconstruction (Fig. 15). In the new implementation, the block geometry only utilizes symmetries in an axial direction. It would be useful to also implement symmetries in the transaxial direction to accelerate the computation.

Since the implementation of block geometry in the STIR library was based on calculating LOR parameters directly from the Cartesian coordinates of detection points, this opens a door to extend the library to more generic geometries, i.e., to any arrangement of the so-called cylindrical PET scanners such that there is no limitation in the size and number of crystals, blocks of crystals, and modules of blocks as long as they stay in a ringed-shape geometry.

## Conclusion

We implemented a more accurate scanner model for PET image reconstruction in the STIR library. Scanner geometries with the shape of a regular polygon can be modeled using the new implementation. The new implementation was tested and is available for use via the ETH library [23]. The new model was evaluated in terms of spatial resolution, partial volume effect, and uniformity using Monte Carlo simulation of the Derenzo and a uniform cylinder phantom. Results indicate a significant improvement for the new model in comparison with the old one which re-sampled the data from a physical polygonal geometry into a virtual cylindrical scanner geometry.

## List of parameters

***f*** (vector): the function of the radiotracer’s distribution

*L* (diagonal matrices): attenuation correction factors

*N* (diagonal matrices): normalization correction factors

***q*** (vector): the mean of the measured data

***r*** (vector): estimate of the mean random events

***s*** (vector): estimate of the mean of scatter events

*X* (matrix): the geometric model that relates the projection space to the image space

## Data Availability

The new implementation for image reconstruction and details of software implementation and installation are available: 10.5905/ethz-1007-146. Simulated data cannot be provided, but the simulation procedure—phantoms and scanner geometry—has been described in the “Monte Carlo simulations” section. Simulated data of any scanner, representable by the above-described block-type implementation in STIR, can be used to replicate the results and validate the findings, namely demonstrate the improved image quality, due to the proper representation of the scanner geometry.

## Abbreviations

COV: Coefficient of variation
ETH: Swiss Federal Institute of Technology in Zurich
FOV: Field of view
FWHM: Full width half maximum
LOR: Line of response
MPI: Message Passing Interface
MRI: Magnetic resonance imaging
PET: Positron-emission tomography
PTV: Peak-to-valley
RC: Recovery coefficient
ROI: Region of interest
SAFIR: Small Animal Fast Insert for MRI
SOR: Spill-over ratio
STIR: Software for Tomographic Image Reconstruction
